# Protocol and Preliminary Results of the Establishment of Intracranial Aneurysm Database for Artificial Intelligence Application Based on CTA Images

**DOI:** 10.3389/fneur.2022.932933

**Published:** 2022-07-19

**Authors:** Wei You, Yong Sun, Junqiang Feng, Zhiliang Wang, Lin Li, Xiheng Chen, Jian Lv, Yudi Tang, Dingwei Deng, Dachao Wei, Siming Gui, Xinke Liu, Peng Liu, Hengwei Jin, Huijian Ge, Yanling Zhang

**Affiliations:** ^1^Department of Interventional Neuroradiology, Beijing Neurosurgical Institute and Beijing Tiantan Hospital, Capital Medical University, Beijing, China; ^2^Department of Neurointerventional Engineering and Technology, Beijing Engineering Research Center, Beijing, China; ^3^Department of Neurosurgery, The First People's Hospital of Lianyungang and The First Affiliated Hospital of Kangda College, Nanjing Medical University, Lianyungang, China; ^4^Department of Neurosurgery, The People's Hospital of Nanpi Country, Cangzhou, China; ^5^Department of Emergency, The Fourth Hospital of Hebei Medical University, Shijiazhuang, China; ^6^Department of Roradiology, Beijing Tiantan Hospital, Capital Medical University, Beijing, China

**Keywords:** intracranial aneurysm, database, artificial intelligence, computed tomographic angiography, deep learning

## Abstract

**Background and Purpose:**

Unruptured intracranial aneurysms (UIAs) are increasingly being detected in clinical practice. Artificial intelligence (AI) has been increasingly used to assist diagnostic techniques and shows encouraging prospects. In this study, we reported the protocol and preliminary results of the establishment of an intracranial aneurysm database for AI application based on computed tomography angiography (CTA) images.

**Methods:**

Through a review of picture archiving and communication systems, we collected CTA images of patients with aneurysms between January 2010 and March 2021. The radiologists performed manual segmentation of all diagnosed aneurysms on subtraction CTA as the basis for automatic aneurysm segmentation. Then, AI will be applied to two stages of aneurysm treatment, namely, automatic aneurysm detection and segmentation model based on the CTA image and the aneurysm risk prediction model.

**Results:**

Three medical centers have been included in this study so far. A total of 3,190 cases of CTA examinations with 4,124 aneurysms were included in the database. All identified aneurysms from CTA images that enrolled in this study were manually segmented on subtraction CTA by six readers. We developed a structure of 3D-Unet for aneurysm detection and segmentation in CTA images. The algorithm was developed and tested using a total of 2,272 head CTAs with 2,938 intracranial aneurysms. The recall and false positives per case (FP/case) of this model for detecting aneurysms were 0.964 and 2.01, and the Dice values for aneurysm segmentation were 0.783.

**Conclusion:**

This study introduces the protocol and preliminary results of the establishment of the intracranial aneurysm database for AI applications based on CTA images. The establishment of a multicenter database based on CTA images of intracranial aneurysms is the basis for the application of AI in the diagnosis and treatment of aneurysms. In addition to segmentation, AI should have great potential for aneurysm treatment and management in the future.

## Background

Saccular unruptured intracranial aneurysms (UIAs) are pathological artery dilation that occurs in major cerebral arteries branch and affects 3–5% of the adult population (1). Approximately 20–30% of patients with intracranial aneurysms harbor more than one aneurysm (2). With the improvement in the quality of intracranial imaging techniques over the past 20 years and the more widespread application of magnetic resonance imaging (MRI) and computed tomography (CT) as diagnostic tools (3), UIAs are increasingly being detected in clinical practice. A recent cross-sectional study showed that the prevalence of UIA is as high as 7% among individuals aged 35–75 years in China (4). Subarachnoid hemorrhage caused by aneurysm rupture has serious consequences, with the mortality rate of early hemorrhage being 40%, and the rate of rebleeding being as high as 60–70% (5). Surgical clipping or interventional therapy for aneurysms is associated with an inherent risk of invasions, with a 4.3–4.6% incidence of post-operative morbidity and a 10–24.6% incidence of new neurological deficits following treatment (6). Clinicians are increasingly faced with the dilemma of choosing appropriate clinical management, either prophylactic treatment (endovascular or aneurysm clipping) with an inherent risk of complications or conservative treatment that leaves patients at risk of aneurysm rupture. Establishing a reliable model to determine the stability of UIAs is important for therapeutic decisions in unruptured aneurysms.

## Imaging Diagnostic Tools for Intracranial Aneurysms

The detection and risk assessment of intracranial aneurysms is critical due to their low rupture rate and high rates of disability and mortality after rupture. IAs are most often detected incidentally after the rupture of aneurysms or during the evaluation of systemic symptoms, such as headache, neural paralysis, and ischemic cerebrovascular disease. At present, computed tomography angiography (CTA), magnetic resonance angiography (MRA), and digital subtraction angiography (DSA) are the main imaging diagnostic tools for intracranial aneurysms. Each tool has advantages and disadvantages, and different individual practitioners use them variably at various stages in the evaluation of an aneurysm.

As the “gold standard” for aneurysm diagnosis, DSA has shown greater sensitivity, especially in aneurysms smaller than 3 mm (7, 8). However, it is difficult to become a large-scale disease screening method due to its inherent invasiveness. MRA for aneurysm imaging uses contrast methods or time-of-flight (TOF) sequences. In general, MRA has been reported to have a detection sensitivity ranging from 74 to 98% (9). However, a study showed that the sensitivity of MRA can be significantly affected by the aneurysm size. Therefore, magnetic resonance was suggested as a primary method of screening for UIAs and can be very useful for aneurysms larger than 3 mm. Possible complications for DSA include contrast agent allergy events, IA rupture, brain infarction, and arterial injury (10). With the development of multidetector scanners, CTA is frequently added to assist in the diagnosis of the aneurysm. In general, the sensitivity, specificity, and accuracy of aneurysm detection are very high compared with DSA with the 3D rotational acquisition, with 1 report indicating values of 96.3, 100, and 94.6%, respectively (11). Even for smaller aneurysms (typically for those <3 mm), there was a comparable sensitivity, specificity, and accuracy (81.8, 100, and 93.3%, respectively). Therefore, with its high sensitivity and specificity, CTA can be considered an initial diagnostic test for aneurysm detection and screening (12).

### Management of UIAs and Predictors of Rupture

After a UIA is detected, several factors must be considered to determine the appropriate management approach. Despite the continuous improvement of surgical clipping and endovascular treatment techniques, the therapeutic effect of the intracranial aneurysm has been gradually improved, but there are still certain complications and morbidity mortality in various treatment methods. There are no randomized clinical trial data that define the optimum management of a UIA. The available natural history studies provide both retrospective and prospective data. The risk of aneurysm rupture without any intervention should be compared with the risk of surgical clipping or endovascular treatment. The need for preventive treatment of unruptured aneurysms is controversial, especially considering the low rupture rate of intracranial aneurysms, which is only 0.25% (12). Therefore, determining its stability is important to make therapeutic decisions for unruptured aneurysms (13, 14). The most reasonable treatment strategy for intracranial aneurysms may be to screen patients with a high risk of rupture and carry out active intervention treatment and to carry out conservative and follow-up treatment for patients with a low risk of rupture.

Many studies have identified important clinical factors for aneurysm rupture. Size is proposed as the most important factor for predicting aneurysm stability in previous studies. Up to 85–90% of ruptured intracranial aneurysms are <10 mm in diameter (15–17). According to the International Study of Unruptured intracranial aneurysms, aneurysms of <7 mm in the anterior and posterior circulation have a cumulative 5-year risk of rupture of 0 and 2.5%, respectively (18). However, a published lifelong follow-up study that smoking and female gender were more severe prognostic factors than aneurysm size (19). Old age was also determined as a risk factor for aneurysm rupture in a prospective study (20). As growing aneurysms are at high risk for rupture compared with stable size and stable shape (21), discriminating aneurysm stability is meaningful. In addition to size and patient's baseline information, the morphology of the aneurysm is closely related to the stability of the aneurysm (13, 14). Some studies have found that various morphological features of aneurysms, such as size ration, flow angle, height/width ratio, aspect ratio, and deviated angle, are associated with their rupture. However, these parameters are mainly measured in a two-dimensional projection and may differ among different angle projections or measurers.

It is well-known that three-dimensional images contain primitive and comprehensive morphological information, and radiomics and machine learning studies based on these images have attempted to identify important morphological features associated with aneurysm stability. In a study containing 719 aneurysms, the researchers used radiomics to find that SphericalDisproportion, Maximum 2D diameter slice, and surface area were the most important morphological feature to predict aneurysm stability (22). However, the research on these complex three-dimensional parameters is still incomplete, and there are obvious problems, such as insufficient scientific research data and inconsistent experimental results. Their values of aneurysm rupture risk assessment and prediction need to be further verified (14, 23).

Therefore, it is urgent to explore the risk factors closely related to intracranial aneurysm rupture that is convenient for quantitative analysis and to establish an accurate and effective risk assessment system for intracranial aneurysm rupture on this basis, providing a reliable basis for treatment decisions, which has great clinical and social value.

### Electronic Medical Data

At present, almost all tertiary medical centers in China use electronic medical record systems and picture archiving and communication system (PACS). Digitization of the medical records and the storage of image data in the “DICOM” format provides an opportunity to utilize medical data for standardization and analysis in unique ways. In addition, modern tools used for extraction from electronic medical records and image data from PACS can be used to develop a database that can be used for retrospective studies and registry studies and can even be used for establishing stratified disease models. Digitization of medical data makes it possible to analyze imaging data of a large number of patients based on medical records. Although there are a large number of patients with intracranial aneurysms and a large amount of relevant medical data in medical centers in China, there are currently data barriers between hospitals, making data sharing difficult. The establishment of a multicenter database is the foundation for high-quality, large-scale disease research in the future.

### Artificial Intelligence

The promise of artificial intelligence (AI) in medicine and healthcare is widely discussed at present, just as it was when the concept was first proposed in the late 1960s. AI focuses exclusively on the use of algorithms and the software that implements them to approach human cognition abilities to analyze complex observations and data. AI works by modeling and extracting information from various data sources and then processing it, with the goal of being able to provide a well-defined, ideally understandable, and interpretable output to the practicing medical professional. It encompasses different analytical methods, such as machine learning (ML), natural language processing, and computer or machine vision (24). ML, represented by deep learning, represents the most successful branch of AI, focusing on the development of programs with the ability to learn from data (25).

It has recently been used to aid in diagnostic techniques, and several studies have attempted to apply ML methods to neuroimaging data to assist in stroke diagnosis (26). The relevant published literature has focused on several disease types, namely, cancer, neurological diseases, and cardiovascular diseases (26). The application of AI relies on massive amounts of medical data being collected and stored in the form of electronic medical records, especially including rich medical imaging information. A major advantage of deep learning is the convolutional neural network (CNN), which analyzes pixel-level information in images and is able to interpret the orientation of pixels, which allows them to identify lines, curves, and even objects in images. A study shows that the applications of AI in precision oncology are due in large part to its remarkable ability to classify imaging data across different clinical domains (25). Data mining has great significance for identifying new diagnostic markers that can precisely diagnose and classify diseases, such as intracranial aneurysms, based on information about them.

## Methods and Design

### Building an IA Database Based on the CTA Image for AI

We aim to apply the full advantage of AI to the diagnosis and treatment of aneurysms, including automatic diagnosis of aneurysms, automatic segmentation, and risk assessment of aneurysms. Therefore, we will establish a multicenter database based on CTA images of intracranial aneurysms, which is the basic work of automatic aneurysm diagnosis, segmentation, and aneurysm rupture risk prediction based on AI technology. To construct the database, previous retrospective data and prospective data enrolled during the registration of studies will be included.

The reason why we are willing to explore this issue is due to the urgent clinical needs: (1) compared with the limited physician resources, there are a large number of aneurysm images to be diagnosed; (2) more reliable aneurysm risk assessment methods are needed to develop reasonable treatment strategies. Also at the technological frontier, we are seeing clear reasons, including data availability and expert opinion, that this exploration is feasible.

**Figure 1:**
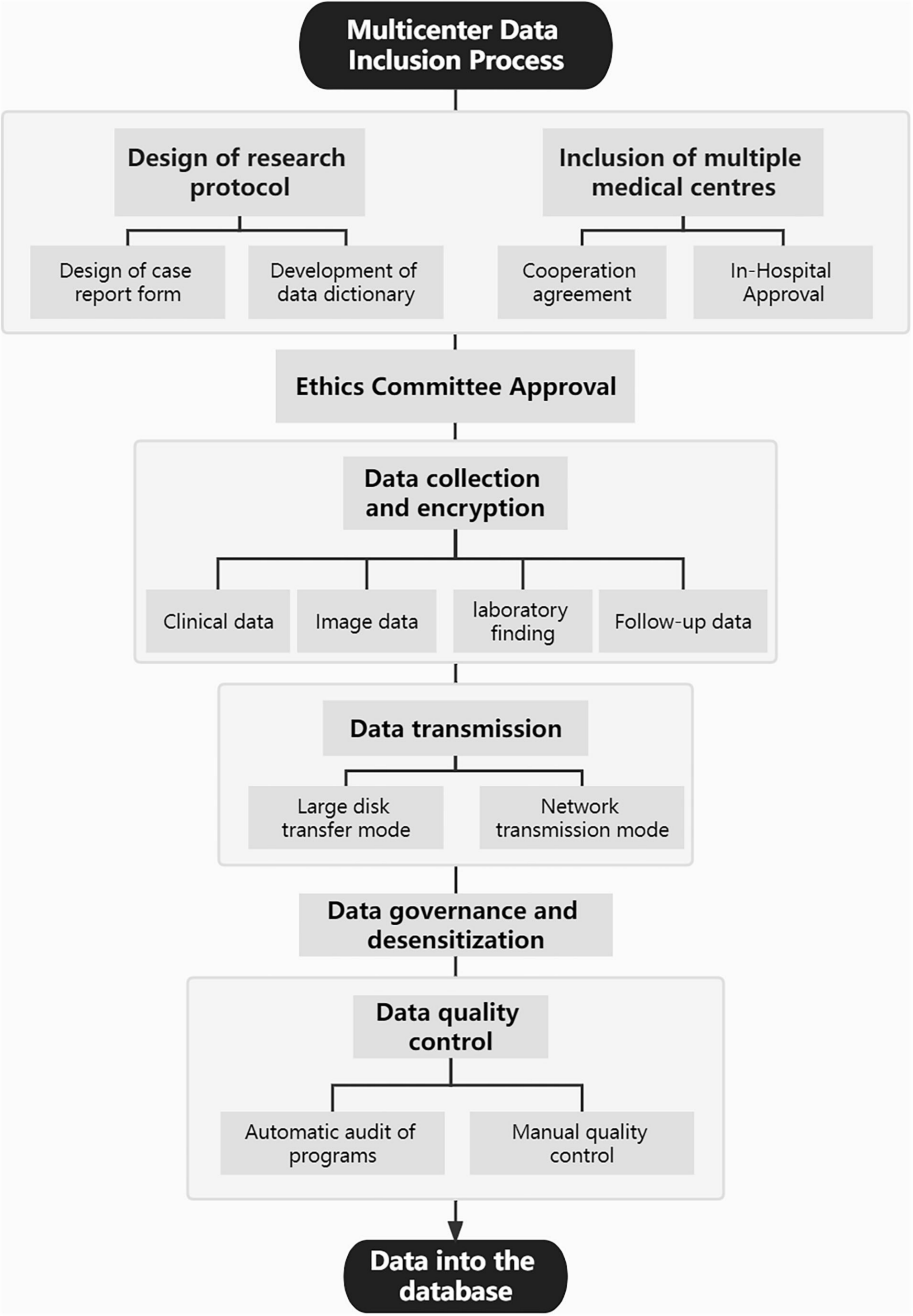
The process of multicenter data inclusion.

This study was approved by the medical ethics committee of Tiantan Hospital, Beijing, China. Through a review of the picture archiving and communication system in Tiantan hospital, we collected CTA images of all patients with aneurysms treated in our hospital between January 2010 and March 2021. In addition, CTA data of patients with aneurysms from two other Chinese medical centers were also collected. Furthermore, on the basis of all enrolled patients with aneurysms with CTA images, the corresponding clinical data were collected through the electronic medical record systems, including basic clinical characteristics, relevant data concerning personal and family history, relevant laboratory findings, and follow-up information. Figure 1 shows the multicenter data inclusion process. Data continue to be added through observational registries (registration number: ChiCTR2100054564). Patients' treatment was not affected by their participation in this study.

### Recruitment of Participants

#### Collection Criteria

At least one UIA that has not been treated and has been confirmed by imaging: CTA, MRA, and/or DSA.

Complete CTA image sequence and clinical data.

Patients or relatives agreed to participate in this study.

#### Exclusion Criteria

Computed tomography angiography with arteriovenous malformation, arteriovenous fistula, or Moyamoya disease.

Post-traumatic or infectious pseudoaneurysm.

Previous surgical clipping or endovascular treatment for the aneurysm.

Non-diagnostic image quality with severe artifacts as judged by an attending neuroradiologist.

Incomplete or missing CTA imaging data and clinical data.

### Manual Segmentation of Aneurysm for Model Training

**Figure 2:**
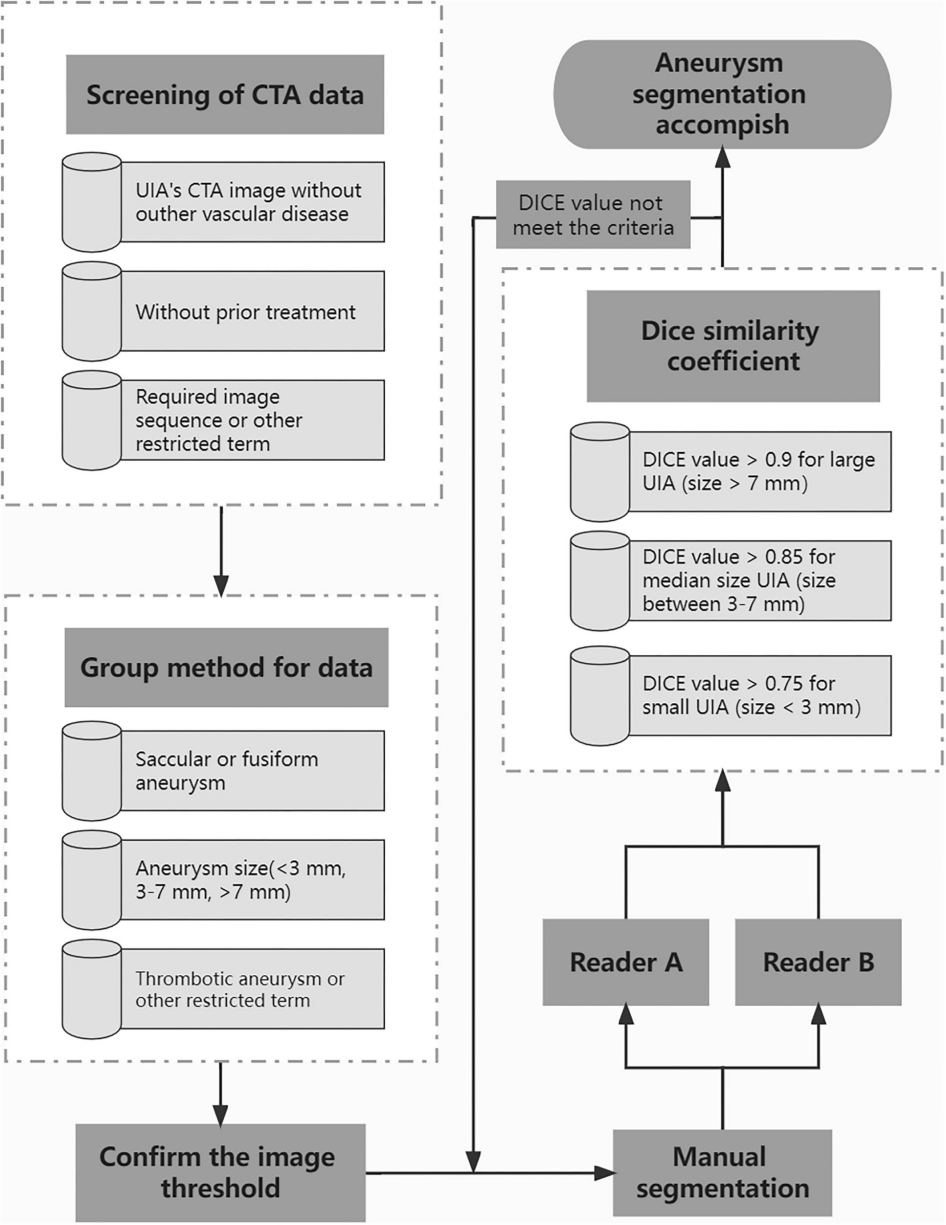
The standard procedure for manual aneurysm segmentation.

A standard procedure for manual aneurysm segmentation (Figure 2) was developed through consultation between doctors and technical engineers, and the test of the standard was completed through a small amount of data. All operators were trained in a standardized manner prior to formal manual segmentation of aneurysms. Eligible CTA data will be grouped on each slice on the basis of aneurysm characteristics. Identified aneurysms were manually segmented on subtraction CTA by radiologists using 3D Slicer (version 4.10.1). Each data will be randomly assigned to two readers at the same time for segmentation using the same threshold. Dice similarity coefficient (DSC) was used to evaluate the annotation performance of two readers. For the aneurysm sizes >7, 3–7, and <3 mm, the DSC criteria for segmentation were >0.9, >0.85, and >0.75, respectively. If the DSC does not meet the criteria, it will be returned for re-annotation until meets the requirement.

### Aneurysm Detection and Segmentation Model

All data included in this study were divided into training, validation, and test datasets in a ratio of 7:1.5:1.5. The training dataset was used to develop the 3D-Unet model, the validation dataset was used to validate the algorithm and adjust the model hyperparameters, and the test dataset was used to evaluate the generalization ability of the model. Manual aneurysm detection and segmentation were used as the reference standard to evaluate the performance of the final model.

For the study on algorithm development, we developed a structure of 3D-Unet for aneurysm segmentation in CTA images. The network takes CTA as input and outputs a probability mask, which contains the probability of whether each voxel belongs to the aneurysm. Each CTA image was cropped into patches with the same size of (32, 224, 224) for training the 3D-Unet. Hounsfield units of each patch image were normalized to (0, 1). Data augmentations methods, such as random crop and random zoom, were used for robust training of the network. The total loss was the sum of the losses of the main output and module and minimized using the Adam Optimizer.

The development of automated detection models for aneurysms has the potential to reduce reading time and increase radiologists' performance in laboratory and clinical environments. The detection model may also benefit patients undergoing CTA (those with a headache or acute ischemic stroke) because it may reduce the likelihood of an incidental aneurysm being undetected. Additionally, the realization of automatic segmentation of aneurysms will establish a good foundation for the risk prediction model in the next stage. It can provide a large amount of learnable data for risk prediction models in learning aneurysm morphological features in a short time, breaking through the limitation of the high labor cost of manual segmentation. Furthermore, it can reduce observer variability and avoid bias between observers.

### Risk Prediction Models for Aneurysms

The risk of IA can be divided into three stage-related factors: the risk of aneurysm development, the risk of growth or morphological changes, and the risk of rupture. Among them, we focused on the risk of growth and rupture following aneurysm development. We divided all aneurysm data into the stable group and unstable group based on morphological changes of aneurysms under multiple imaging follow-up examinations and aneurysm hemorrhage. Aneurysm data in the unstable group included ruptured and growing aneurysms, which are at high risk. The risk prediction of aneurysms is a complex problem, and the related predictive factors reported in the literature mainly include patient clinical factors, aneurysm morphological parameters, and hemodynamic parameters. Using statistical models to build a multidimensional model for UIA risk prediction that integrates clinical, image, and hemodynamic data is urgently needed in clinical practice. In this process, the deep involvement of AI is the key to the effective use of massive data and the establishment of efficient and accurate models. AI technology will be used for the automatic calculation of morphological parameters based on the automatic segmentation of aneurysms. AI can also use to automatically extract valuable clinical information from a large amount of chaotic clinical data. Additionally, it can realize the rapid and automatic extraction of the hemodynamic parameters of the aneurysm through a large amount of learning of the computational fluid dynamics (CFD) analysis data of the aneurysm. The availability of expert annotations makes this a classic supervised ML problem, although we will also consider exploring semi-supervised and unsupervised learning methods as we continue to add data.

### Statistical Analysis

Normality assumptions were assessed using the Kolmogorov-Smirnoff test. Data are presented as frequencies for categorical variables and means and ranges for continuous variables. Pearson's chi-square test or Fisher's exact test were used to evaluate the differences between ordinal and categorical variables. Independent sample tests were used to examine group differences of continuous variables. A two-sided *P*-value < 0.05 was considered statistically significant. To evaluate the performance of the automatic segmentation model, results were expressed as Dice values. To evaluate the performance of automatic detection, the recall and false positives per case (FPs/case) of all aneurysms were evaluated in the test dataset. Multiple regression analysis will be used to calculate OR value for high-risk factors or significant parameters. A multidimensional model for UIA risk prediction will be used to assess the risk of aneurysm rupture and growth in the prospective cohort group and thus determine the model's accuracy and efficacy. Statistical analysis was performed using SPSS 22 (IBM, Chicago, IL, USA), and figures were generated using GraphPad Prism (GraphPad Software, San Diego, CA, USA).

## Results

### Data Inclusion

Three medical centers, including Beijing Tiantan Hospital, have been included in this study so far. In a retrospective review of the picture archiving and communication systems at Beijing Tiantan Hospital from January 2010 to March 2021, CT data of a total of 7,855 patients with radiologically reported intracranial aneurysms were obtained. Among 2,896 CT without CTA, 1,671 CT was not from the head scan, 66 CTA was with arteriovenous malformation, fistula, and moyamoya disease, 230 CTA showed with previous treatment, and 11 CTA was with poor image quality with severe artifacts, thus excluded from this database. A total of 2,981 cases of CTA examinations with 3,872 aneurysms were included from Tiantan Hospital in the database. In the same way, a total of 209 CTA examinations with 252 aneurysms enrolled the database from the two other medical centers. In the end, a total of 3,190 CTA examinations with 4,124 aneurysms included in the database. The overview of the characteristics of the database is shown in Table 1. All identified aneurysms from CTA images that enrolled in this study were manually segmented on subtraction CTA by 6 readers, and the results met the criteria for segmentation. Additional medical centers and aneurysms data will continue to be added to the database in the future through the registry study.

**Table 1:** The overview of the characteristics of the database.

**Characteristic**	**Total**
No. of patients, *n*	3,190
No. of IAs, *n*	4,124
Male, *n* (%)	1,219 (38.2)
**Age, years**	56.7 ± 11.0
Male, years	55.8 ± 11.5
Female, years	57.2 ± 10.6
**Aneurysm size, mm**	2.3 ± 0.5
<3 mm, *n* (%)	1,540 (37.3)
3–7 mm, *n* (%)	2,035 (49.3)
>7 mm, *n* (%)	545 (13.2)
**Aneurysm's location**
ICA, *n* (%)	2,384 (57.8)
ACA, *n* (%)	227 (5.5)
ACoA, *n* (%)	403 (9.8)
MCA, *n* (%)	696 (16.9)
PCA, *n* (%)	89 (2.2)
PCoA, *n* (%)	28 (0.7)
BA, *n* (%)	151 (3.7)
VA, *n* (%)	109 (2.6)
Other, *n* (%)	37 (0.9)
**Scanners**
GE healthcare, *n* (%)	2,699 (84.6)
Philips scanners, *n* (%)	482 (15.1)
Other scanner, *n* (%)	9 (0.3)

*ICA, internal carotid artery; ACA, anterior cerebral artery; ACoA, anterior communicating artery; MCA, middle cerebral artery; PCA, posterior cerebral artery; PCoA, posterior communicating artery; BA, basilar artery; VA, vertebral artery*.

### Algorithm Development

At present, we have applied deep learning methods to the diagnosis and segmentation of aneurysms on CTA images. In this part, a total of 2,272 CTA examinations with 2,938 aneurysms were enrolled in this algorithm development. The training dataset contained 1,606 CTA examinations with 2,078 aneurysms, the validation dataset contained 314 CTA examinations with 414 aneurysms, and the test dataset contained 352 CTA examinations with 446 aneurysms. It should be noted that all cases from the test dataset underwent CTA examinations verified by DSA examination within 1 month. The characteristics of each dataset are shown in Table 2.

**Table 2:** The overview of the characteristics of each dataset.

**Characteristic**	**Training set**	**Validation set**	**Test set**	**Total**
No. of patients, *n*	1,606	314	352	2,272
No. of IAs, *n*	2,078	414	446	2,938
CTA with single IA, *n*	1,233	237	283	1,753
CTA with multiple IAs, *n*	373	77	69	519
Female, *n* (%)	1,008 (62.8)	183 (58.3)	216 (61.4)	1,407 (61.9)
**Age, years**	56 ± 10	57 ± 12	55 ± 10	56 ± 11
Male	56 ± 11	57 ± 11	53 ± 10	56 ± 11
Female	57 ± 10	57 ± 12	56 ± 10	57 ± 10
**Aneurysm size, mm**	4.2 ± 3.4	3.6 ± 2.5	6.0 ± 4.6	4.4 ± 3.6
<3 mm, *n* (%)	880 (42.3)	209 (50.5)	94 (21.1)	1,183 (40.3)
3–7 mm, *n* (%)	982 (47.3)	174 (42)	242 (54.3)	1,398 (47.6)
>7 mm, *n* (%)	216 (10.4)	31 (7.5)	110 (24.7)	357 (12.2)
**Aneurysm's location**
ICA, *n* (%)	1,344 (64.7)	258 (62.3)	171 (38.3)	1,773 (60.3)
ACA, *n* (%)	98 (4.7)	21 (5.1)	30 (6.7)	149 (5.1)
ACoA, *n* (%)	143 (6.9)	23 (5.6)	65 (14.6)	231 (7.9)
MCA, *n* (%)	299 (14.4)	65 (15.7)	136 (30.5)	500 (17)
PCA, *n* (%)	50 (2.4)	7 (1.7)	6 (1.3)	63 (2.1)
PCoA, *n* (%)	13 (0.6)	2 (0.5)	10 (2.2)	25 (0.9)
BA, *n* (%)	66 (3.2)	20 (4.8)	11 (2.5)	97 (3.3)
VA, *n* (%)	53 (2.6)	17 (4.1)	14 (3.1)	84 (2.9)
Other, *n* (%)	12 (0.6)	1 (0.2)	3 (0.7)	16 (0.5)
**Scanners**
GE healthcare, *n* (%)	1,601 (99.7)	314 (100)	352 (100)	2,267 (99.8)
Philips scanners, *n* (%)	5 (0.3)	0 (0)	0 (0)	5 (0.2)

*ICA, internal carotid artery; ACA, anterior cerebral artery; ACoA, anterior communicating artery; MCA, middle cerebral artery; PCA, posterior cerebral artery; PCoA, posterior communicating artery; BA, basilar artery; VA, vertebral artery*.

**Figure 3:**
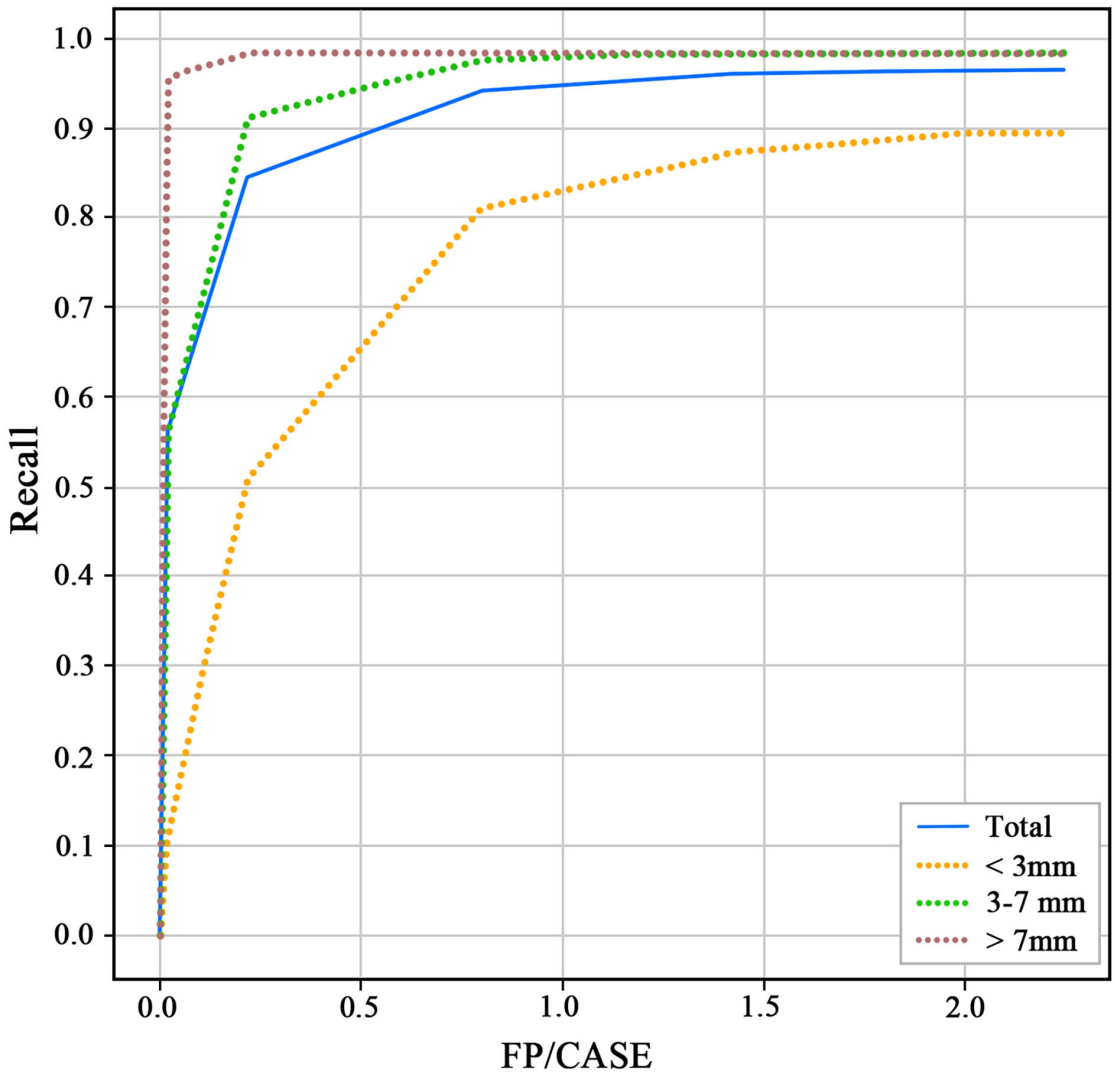
Receiver operating characteristics (ROC) of different size aneurysms for the test dataset.

The 3D-Unet algorithm was developed with the training and validation datasets. After the training procedure, the hyperparameters with the best sensitivity on the validation dataset were chosen. For the test dataset, the recall and FP/case of the model to detect aneurysms were 0.964 and 2.01 (Figure 3). For the aneurysm sizes <3, 3–7, and >7 mm, the recall was 0.894, 0.983, and 0.982, respectively. The Dice similarity coefficient was used to evaluate the model performance of IA segmentation. For the test dataset, the Dice values are 0.783. For the aneurysm sizes <3, 3–7, and >7 mm, the Dice values are 0.635, 0.796, and 0.868, respectively.

## Discussion

Patient's clinical data and relevant image data house the most original and important data. With the improvement of the quality of intracranial imaging technology and the wide application of CT as a diagnostic tool, the clinical radiological examination for neurologic diagnoses is increasing, which requires human expertise in image interpretation. However, there is a relative shortage of experienced radiologists due to the increased demand for imaging diagnoses (27). As a result, there can be uncertainty and inevitable mistakes when making diagnoses and decisions. Large volumes of medical and imaging data are particularly suitable for the application of advanced computing technologies, especially AI and the related field of machine learning. With its high sensitivity and specificity and less invasiveness, CTA has been considered an initial diagnostic test for aneurysm screening and has generated a large amount of data in clinical practice, thus especially suitable for the development of an automatic diagnosis model of the aneurysm.

Our goal is to apply the full advantage of AI to the diagnosis and treatment of aneurysms. Therefore, the establishment of a multicenter database based on CTA images of intracranial aneurysms is the basis for the application of AI in the diagnosis and treatment of aneurysms. The reason why we are willing to explore this issue is due to the urgent clinical needs: (1) compared with the limited physician resources, there are a large number of aneurysm images to be diagnosed; (2) more reliable aneurysm risk assessment methods are needed to develop reasonable treatment strategies. Also at the technological frontier, we are seeing clear reasons, including data availability and expert opinion, that this exploration is feasible.

In addition to segmentation, AI should have great potential for aneurysm treatment and management in the future. Quantitative measurement of aneurysm morphological parameters and prediction of treatment risk and post-operative complications are potential image-based AI applications. In addition to the image data, this study also discusses the clinical baseline data and hemodynamic factors with the aim of making them more applicable to the clinical situation. Additionally, since the study only included Chinese individuals, the results will be more applicable to Chinese people, and the applicability to other populations is unknown.

## Conclusion

A representative database comprising of multicenter based on CTA images of intracranial aneurysms is developed and presented in this work. The database developed comprises the results of segmentation performed by radiologists for each aneurysm, corresponding to the anatomical location and morphological characteristics of the aneurysm. Based on the database, the 3D-Unet algorithm was developed with the recall and FP/case of 0.964 and 2.01 to detect aneurysms and with the Dice values of 0.783 for aneurysm segmentation. In addition to automatic segmentation, AI should have great potential for future aneurysm treatment and management, such as aneurysm growth and rupture risk prediction.

## Data Availability Statement

The raw data supporting the conclusions of this article will be made available by the authors, without undue reservation.

## Ethics Statement

The studies involving human participants were reviewed and approved by Institutional Review Board of Beijing Tiantan Hospital. Written informed consent to participate in this study was provided by the participants' legal guardian/next of kin.

## Author Contributions

WY and YS performed the data analysis and drafted the manuscript. YZ, HG, and XL designed the study. WY, JF, ZW, LL, XC, JL, YT, DD, DW, and SG contributed to data collection. XL, HG, HJ, and PL performed the revision of the current literature. All authors contributed to the article and approved the submitted version.

## Funding

This study has received funding by the National Key Research and Development Program of China (2017YFB1304400), the National Natural Science Foundation of China (81901197 and 82171289).

## Conflict of Interest

The authors declare that the research was conducted in the absence of any commercial or financial relationships that could be construed as a potential conflict of interest.

## Publisher's Note

All claims expressed in this article are solely those of the authors and do not necessarily represent those of their affiliated organizations, or those of the publisher, the editors and the reviewers. Any product that may be evaluated in this article, or claim that may be made by its manufacturer, is not guaranteed or endorsed by the publisher.
